# Supplementary figures and images for: The Mycobacterium tuberculosis PE15/PPE20 complex transports calcium across the outer membrane
Source: PLoS Biol. 2022 Nov 28;20(11):e3001906. doi: 10.1371/journal.pbio.3001906 (PMC9731449; doi:10.1371/journal.pbio.3001906)

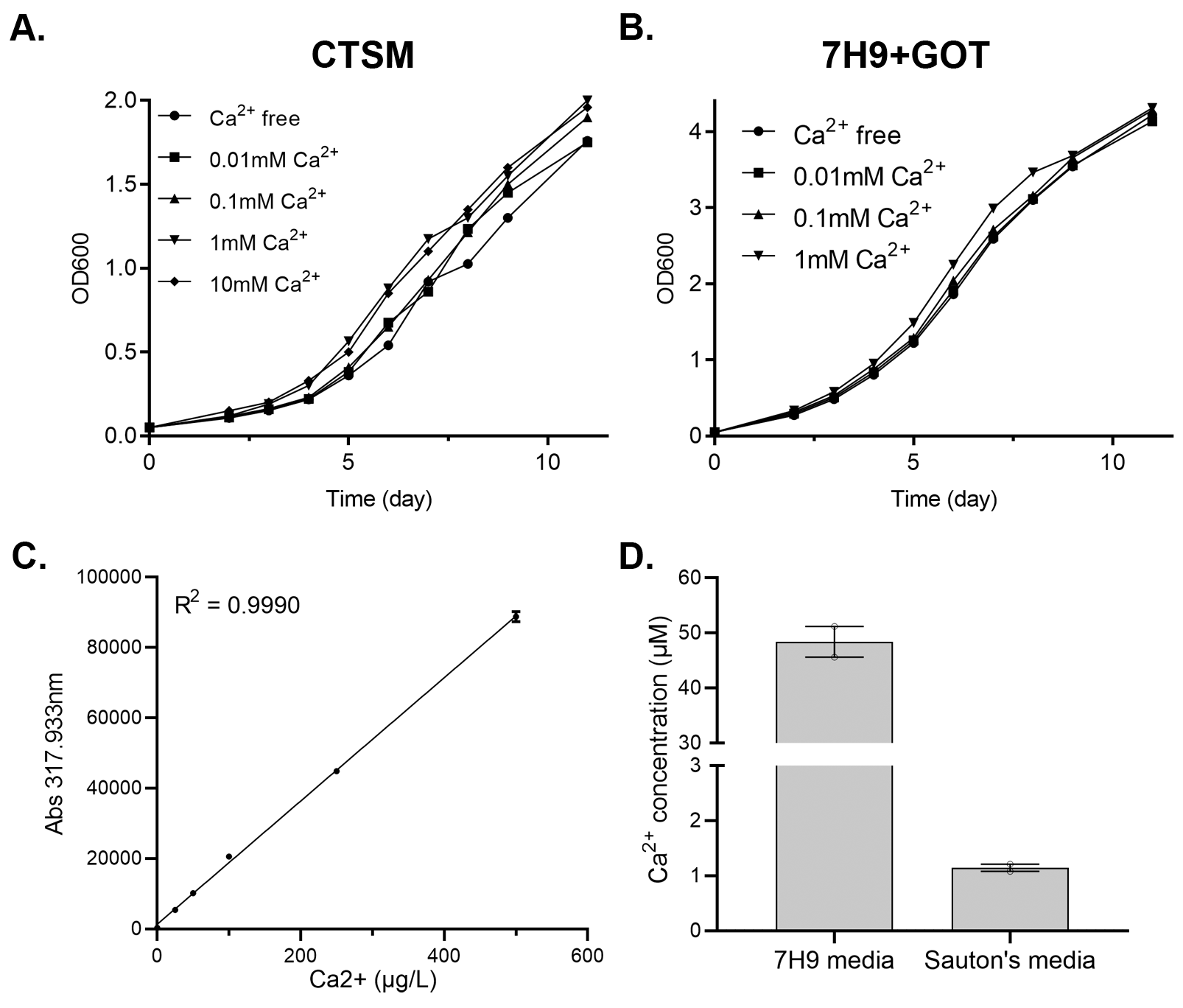

Supplement: S1 Fig — (A) CTSM and (B) 7H9 medium containing glycerol, OADC, and Tween-80 (7H9+GOT) supplemented with different concentrations of CaCl2 shows no effect of Ca2+ on growth. (C) ICP-OES standard curve for the determination of Ca2+ concentrations. (D) Ca2+ concentrations in standard 7H9 and Sauton’s media. The data underlying all the plots in this figure are included in S1 Data. Ca2+, calcium ion; CTSM, Chelex-treated Sauton’s medium; ICP-OES, inductively coupled plasma optical emission spectrometry. (TIF) [file pbio.3001906.s001.tif]

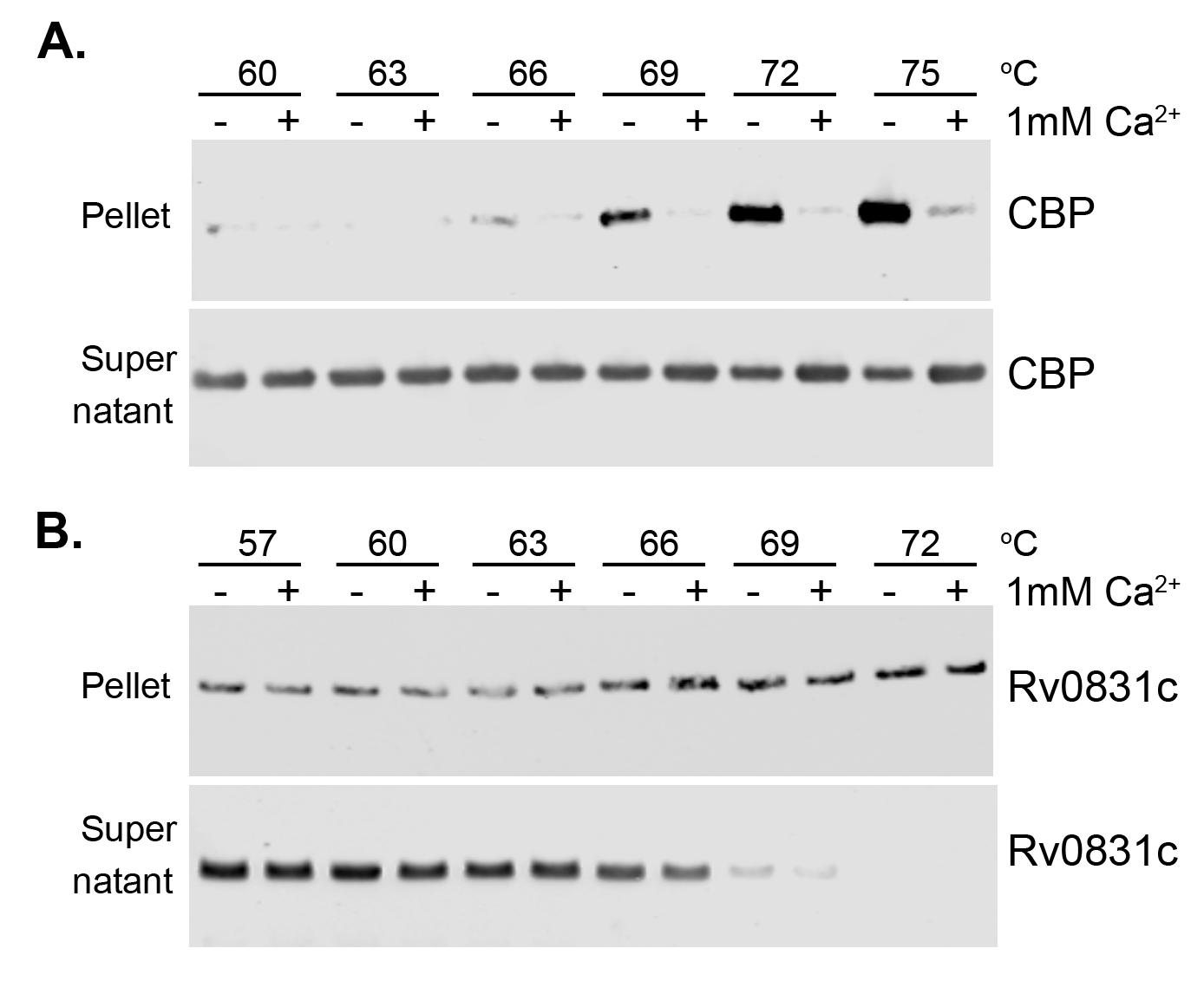

Supplement: S2 Fig — (A) A known CBP from Encephalitozoon cuniculi shows a Ca2+-dependent increase in the melting temperature and concomitant precipitation. (B) A protein not known to bind Ca2+ shows no difference in melting temperature and resulting precipitation. Uncropped images in this figure are included in S1 Raw images. Ca2+, calcium ion; CBP, calcium-binding protein. (TIF) [file pbio.3001906.s002.tif]

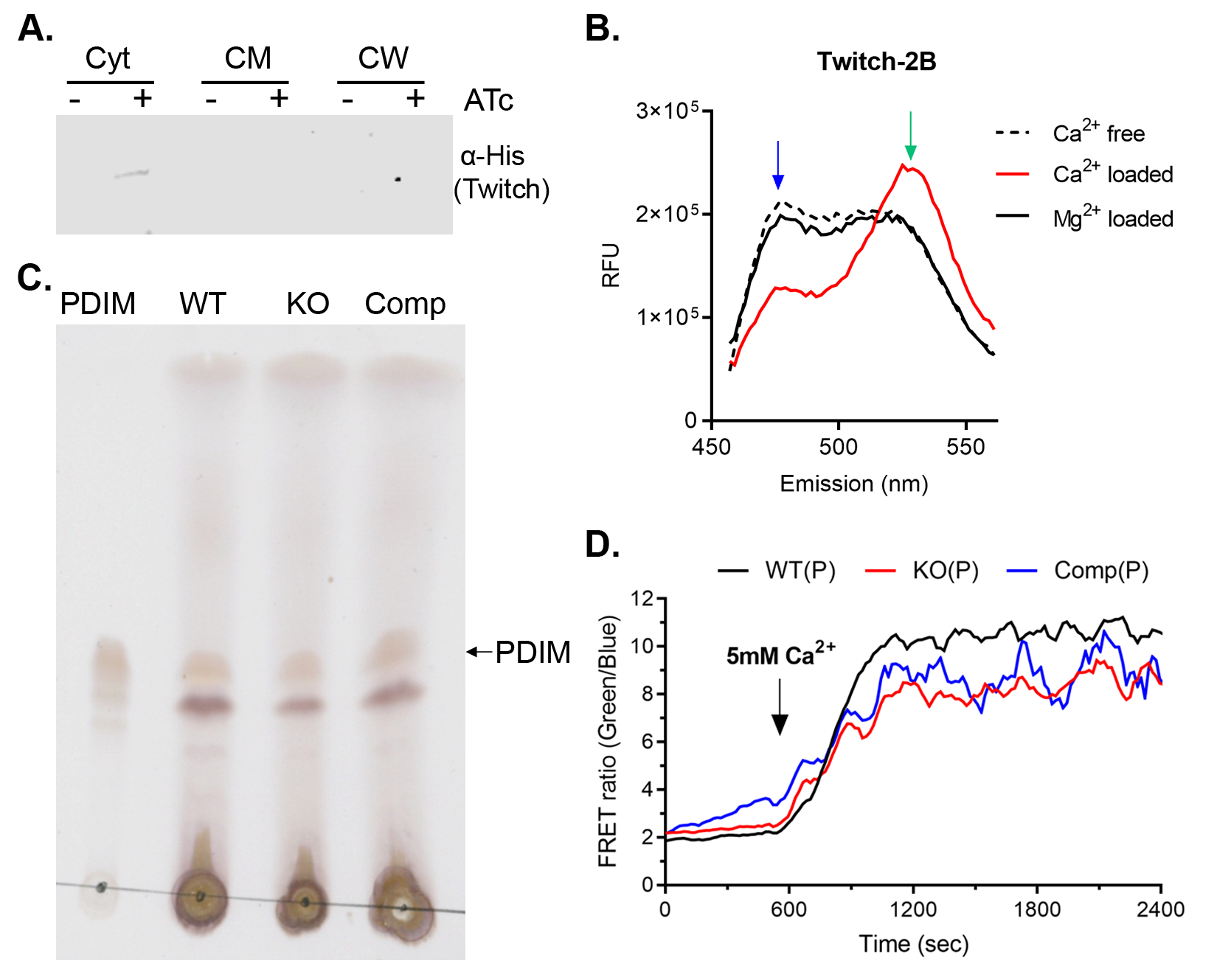

Supplement: S3 Fig — (A) Western blot showing localization of Twitch-2B (His tagged) in the cytosolic fraction. The same cell fractions as in Fig 3A were used, see controls therein. Cyt: cytosolic fraction, CM: cell membrane fraction, CW: cell wall fraction. (B) The FRET signal is highly specific for Ca2+ over the congener Mg2+. The His-tagged Twitch protein was purified from the cytosolic fraction using Ni-NTA column, treated with EDTA and desalted. An emission scan of the purified protein was recorded upon incubation with either Ca2+ or Mg2+. (C) Thin-layer chromatography of the cell wall component PDIM shows no difference in PDIM content between WT and pe15/ppe20 KO strains. (D) Permeabilization of the cell wall diminishes PE15/PPE20’s effect on Ca2+ import. WT, pe15/ppe20 KO, and complemented strain were transformed with Twitch, permeabilized (P) with lysozyme and Triton-X100, and the Ca2+-FRET signal measured. The data underlying all the plots and uncropped images in this figure are included in S1 Data and S1 Raw images. Ca2+, calcium ion; FRET, fluorescence resonance energy transfer; KO, knockout; PDIM, phthiocerol dimycocerosate; WT, wild type. (TIF) [file pbio.3001906.s003.tif]

**Fig. 2A**

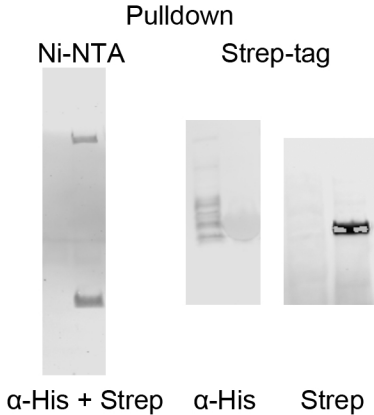

**Fig.2B**

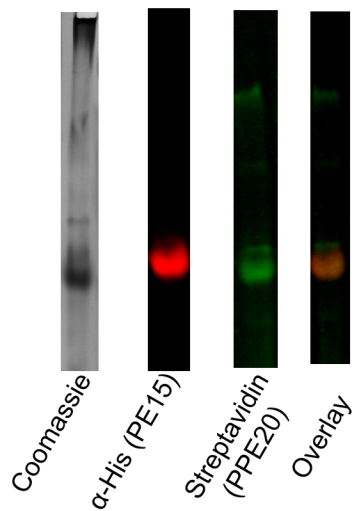

**Fig. 2D**

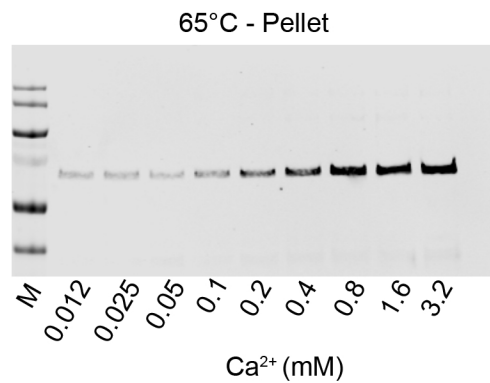

**Fig. 2C**

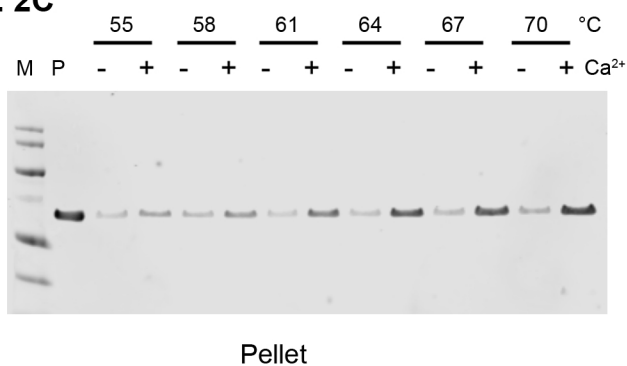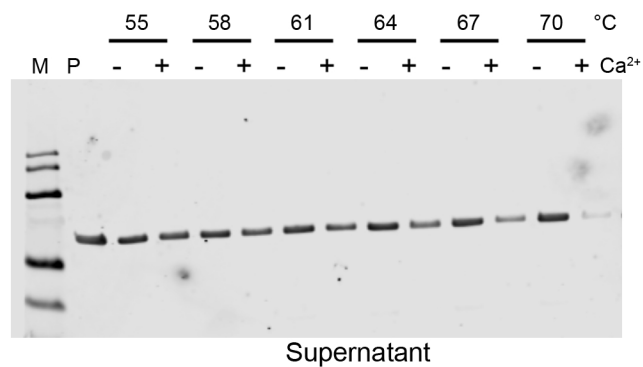

**Fig. 3A and S3A**

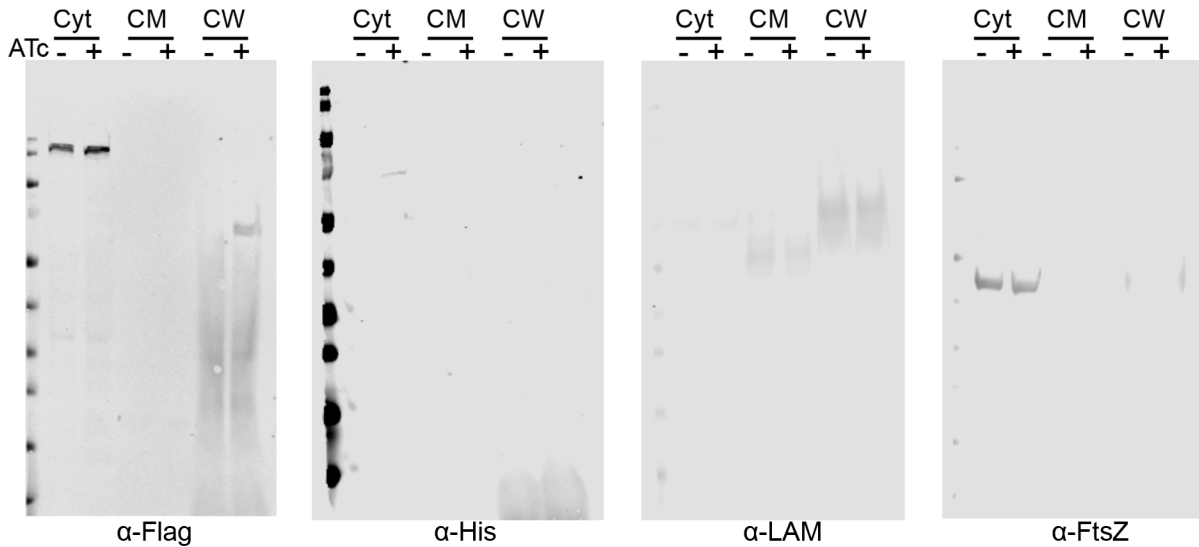

S2A Fig

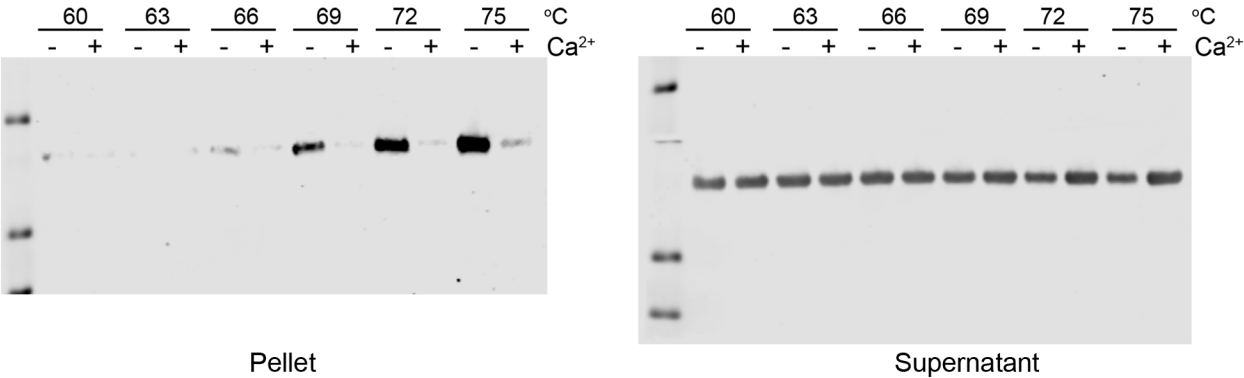

S2B Fig

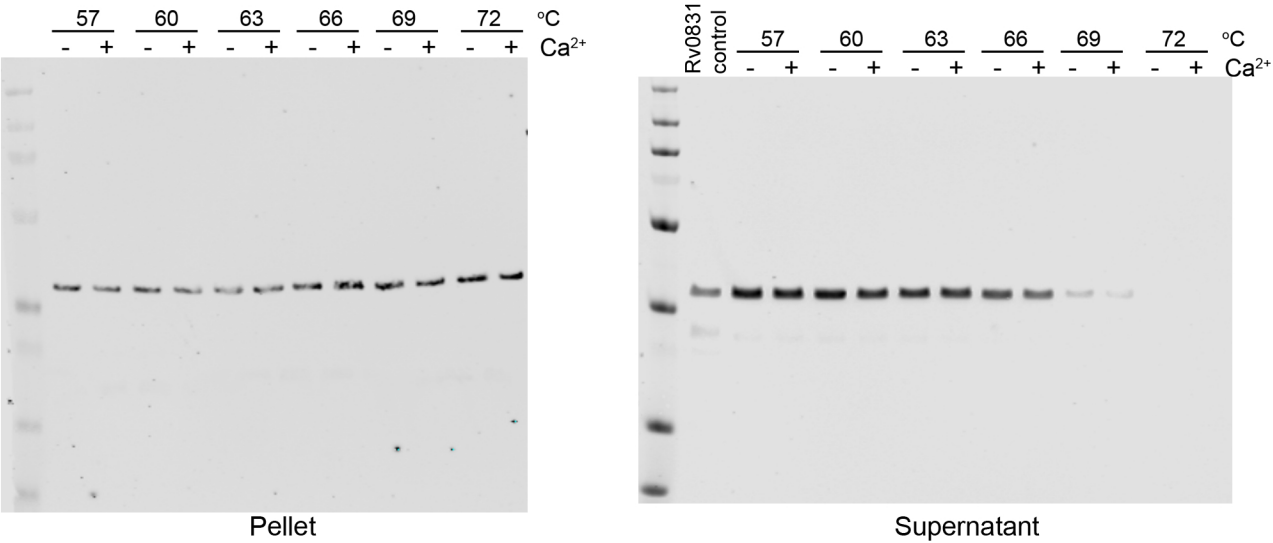

Supplement: S1 Raw images — (PDF) [file pbio.3001906.s006.pdf]
