# Supplementary material for: The Mycobacterium tuberculosis PE15/PPE20 complex transports calcium across the outer membrane
Source: PLoS Biol. 2022 Nov 28;20(11):e3001906. doi: 10.1371/journal.pbio.3001906 (PMC9731449; doi:10.1371/journal.pbio.3001906)
Supplement: S1 Table — (A) Differentially expressed genes in response to Ca2+ in Mtb and (B) primers used in this study. (PDF) [file pbio.3001906.s004.pdf]

S1A Table: Differentially expressed genes in response to Ca^{2+} in *Mtb*

Gene	Symbol	Product	Fold change (log_2)	*P* value
**1hr**				
Rv3269		Conserved protein	0.6059	6E-09
**6hr**				
Rv0253	nirD	nitrite reductase [NAD(P)H] small subunit NirD	-0.8649	3E-12
Rv0252	nirB	nitrite reductase large subunit	-0.8437	1E-16
Rv2428	ahpC	alkyl hydroperoxide reductase subunit C	-0.694	2E-05
Rv1057		Hypothetical protein	-0.6623	9E-06
Rv0706	rplV	50S ribosomal protein L22	0.5922	0.0007
Rv1033c	trcR	two component transcriptional regulator TRCR	0.6411	2E-09
Rv2989		transcriptional regulatory protein	0.6643	0.0067
Rv0653c		TetR family transcriptional regulator	0.6894	3E-06
Rv0839		Hypothetical protein	0.7175	0.0022
Rv0652	rplL	50S ribosomal protein L7/L12	0.7378	2E-06
Rv3848		transmembrane protein	0.7477	0.0028
**Day 1**				
Rv1386	PE15	PE family protein	-0.9997	2E-16
Rv1057		Hypothetical protein	-0.9173	3E-06
Rv1387	PPE20	PPE family protein	-0.9161	5E-10
Rv0211	pckA	phosphoenolpyruvate carboxykinase	-0.873	2E-27
Rv0212c	nadR	transcriptional regulatory protein NadR	-0.8322	3E-22
Rv1144		short-chain type dehydrogenase/reductase	-0.8088	1E-08
Rv3033		Hypothetical protein	-0.7307	3E-10
Rv2590	fadD9	fatty-acid-CoA ligase	-0.715	1E-09
Rv0516c		anti-anti-sigma factor	-0.7128	2E-05
Rv2450c	rpfE	resuscitation-promoting factor RpfE	-0.6887	5E-05
Rv3252c	alkB	transmembrane alkane 1-monooxygenase AlkB	-0.6589	0.0331
Rv2452c		Hypothetical protein	-0.6246	0.0001
Rv1622c	cydB	Cytochrome D ubiquinol oxidase	-0.6146	1E-10
Rv2453c	mobA	molybdopterin-guanine dinucleotide biosynthesis protein A	-0.608	2E-06
Rv3643		Hypothetical protein	-0.591	0.0285
Rv2862A	vapB23	Antitoxin	0.5852	0.0221

Rv0707	rpsC	30S ribosomal protein S3	0.5858	7E-09
Rv2052c		Hypothetical protein	0.5901	7E-13
Rv2927c		Hypothetical protein	0.5911	2E-17
Rv1158c		Hypothetical protein	0.5927	2E-07
Rv2165c	mraW	S-adenosyl-methyltransferase MraW	0.6117	2E-09
Rv0352	dnaJ1	chaperone protein DnaJ1	0.6188	2E-06
Rv2244	acpM	acyl carrier protein	0.623	0.0001
Rv3414c	sigD	RNA polymerase sigma factor SigD	0.6324	2E-17
Rv0710	rpsQ	30S ribosomal protein S17	0.6331	7E-18
Rv0350	dnaK	molecular chaperone DnaK	0.6334	4E-09
Rv2245	kasA	3-oxoacyl-(acyl carrier protein) synthase II	0.6338	0.0009
Rv0033	acpA	acyl carrier protein AcpA	0.635	0.0011
Rv3417c	groEL1	chaperonin GroEL	0.6373	2E-17
Rv1754c		Hypothetical protein	0.6482	9E-14
Rv3267		Hypothetical protein	0.6486	6E-26
Rv2592c	ruvB	Holliday junction DNA helicase RuvB	0.6661	9E-12
Rv0040c	mtc28	secreted proline rich protein MTC28 (proline rich 28 kDa antigen)	0.6668	1E-12
Rv0312		Hypothetical protein	0.667	2E-21
Rv0032	bioF2	8-amino-7-oxononanoate synthase BioF2	0.6748	9E-05
Rv1697		Hypothetical protein	0.6776	6E-27
Rv2594c	ruvC	Holliday junction resolvase	0.6782	1E-11
Rv3588c	canB	carbonic anhydrase	0.6871	3E-14
Rv2747	argA	N-acetylglutamate synthase	0.6899	6E-10
Rv0440	groEL2	chaperonin GroEL	0.7116	2E-09
Rv1435c		proline, glycine, valine-rich secreted protein	0.7143	6E-15
Rv2748c	ftsK	cell division transmembrane protein FtsK	0.7152	5E-26
Rv2846c	efpA	integral membrane efflux protein EfpA	0.7206	4E-12
Rv1285	cysD	sulfate adenylyltransferase subunit 2	0.73	4E-09
Rv0351	grpE	GRPE protein (HSP-70 cofactor)	0.7315	1E-08
Rv1477	ripA	invasion protein	0.7369	9E-29
Rv2398c	cysW	sulfate-transport integral membrane protein ABC transporter CysW	0.7582	8E-18
Rv2243	fabD	acyl-carrier-protein S-malonyltransferase	0.7658	1E-06
Rv0653c		TetR family transcriptional regulator	0.7828	5E-20
Rv1478		invasion protein	0.7856	1E-21
Rv2525c		Hypothetical protein	0.7983	7E-21
Rv2864c		penicillin-binding lipoprotein	0.8026	2E-15

Rv0652	rplL	50S ribosomal protein L7/L12	0.8592	4E-09
Rv1883c		Hypothetical protein	0.8648	9E-43
Rv1816		transcriptional regulatory protein	0.8664	1E-28
Rv3269		Hypothetical protein	0.9156	1E-19
Rv1884c	rpfC	resuscitation-promoting factor RpfC	0.9711	2E-44
Rv0651	rplJ	50S ribosomal protein L10	1.1021	3E-22
Rv1815			1.1704	1E-58
**Day 3**				
Rv1386	PE15	PE family protein	-1.6401	2E-12
Rv1387	PPE20	PPE family protein	-1.2626	4E-37
Rv3094c		Hypothetical protein	-1.2532	1E-23
Rv2087		Hypothetical protein	-1.1364	0.0004
Rv0280	PPE3	PPE family protein	-1.1356	8E-15
Rv3660c		Hypothetical protein	-1.1274	8E-09
Rv3905c	esxF	ESAT-6 like protein	-0.9347	0.0007
Rv2947c	pks15	polyketide synthase PKS15	-0.9253	0.0007
Rv2393	che1	Ferrochelatase	-0.8902	4E-08
Rv2391	sirA	ferredoxin-dependent nitrite reductase NIRA	-0.8752	2E-21
Rv0094c		Hypothetical protein	-0.8738	0.0021
Rv2392	cysH	phosphoadenosine phosphosulfate reductase	-0.8736	1E-19
Rv3467		Hypothetical protein	-0.8654	0.0022
Rv3093c		oxidoreductase	-0.8641	2E-09
Rv2990c		Hypothetical protein	-0.8639	2E-22
Rv2811		Hypothetical protein	-0.8618	0.0005
Rv0962c	lprP	lipoprotein LprP	-0.8362	0.0003
Rv3383c	idsB	polyprenyl synthetase IdsB	-0.8354	4E-08
Rv2590	fadD9	fatty-acid-CoA ligase	-0.8241	5E-14
Rv2591	PE_PGRS44	PE-PGRS family protein	-0.7964	5E-10
Rv1057		Hypothetical protein	-0.7925	1E-09
Rv2948c	fadD22	acyl-CoA synthetase	-0.756	5E-09
Rv2987c	leuD	isopropylmalate isomerase small subunit	-0.7439	4E-08
Rv0397		13E12 repeat family protein	-0.7423	0.002
Rv3123		Hypothetical protein	-0.7394	0.0002
Rv3022A	PE29	PE family protein	-0.733	0.0434
Rv2836c	dinF	DNA-damage-inducible protein F	-0.7301	0.0014
Rv3771c		Hypothetical protein	-0.6913	0.0116
Rv3175		amidase	-0.6751	2E-05
Rv3656c		Hypothetical protein	-0.675	0.0012
Rv3095		putative transcriptional regulatory protein	-0.6696	2E-07

Rv3382c	lytB1	LYTB-like protein LYTB1	-0.6531	4E-07
Rv0840c	pip	proline iminopeptidase	-0.6498	2E-09
Rv0658c		integral membrane protein	-0.6439	0.0009
Rv0921		resolvase	-0.643	0.0287
Rv3659c		Putative uncharacterized protein	-0.6415	6E-05
Rv3904c	esxE	Putative ESAT-6-like protein 12	-0.6379	0.0125
Rv3485c		short chain dehydrogenase	-0.6358	4E-08
Rv3611		Hypothetical protein	-0.6294	0.0143
Rv3595c	PE_PGRS59	PE-PGRS family protein	-0.6284	5E-05
Rv1087	PE_PGRS21	PE-PGRS family protein	-0.6253	0.0015
Rv3919c	gid	16S rRNA methyltransferase GidB	-0.6058	3E-07
Rv2478c		Single-stranded DNA-binding protein	-0.6041	0.0017
Rv3505	fadE27	acyl-CoA dehydrogenase FADE27	-0.6037	0.0356
Rv1587c		REP13E12 repeat-containing protein	-0.5975	8E-05
Rv3657c		Alanine rich membrane protein	-0.5955	0.0055
Rv2584c	apt	adenine phosphoribosyltransferase	-0.5951	0.0074
Rv2331		Hypothetical protein	-0.5924	1E-04
Rv3335c		integral membrane protein	-0.5921	0.0042
Rv3582c	ispD	2-C-methyl-D-erythritol 4-phosphate cytidylyltransferase	-0.589	0.0055
Rv2543	lppA	lipoprotein LppA	-0.587	7E-05
Rv0097		oxidoreductase	0.6061	1E-08
Rv0652	rplL	50S ribosomal protein L7/L12	0.6285	2E-05
Rv0034		Hypothetical protein	0.6288	0.0006
Rv1754c		Hypothetical protein	0.6298	3E-06
Rv1816		transcriptional regulatory protein	0.6303	3E-09
Rv3914	trxC	thioredoxin trxC (TRX) (MPT46)	0.6346	2E-06
Rv1284	canA	Beta-carbonic anhydrase	0.6363	0.0009
Rv3117	cysA3	thiosulfate sulfurtransferase CysA3	0.6465	8E-08
Rv0815c	cysA2	thiosulfate sulfurtransferase CysA2	0.6516	7E-08
Rv2271		Hypothetical protein	0.6539	4E-06
Rv0651	rplJ	50S ribosomal protein L10	0.6636	2E-06
Rv3269		Hypothetical protein	0.6735	2E-06
Rv0032	bioF2	8-amino-7-oxononanoate synthase BioF2	0.71	7E-09
Rv1884c	rpfC	resuscitation-promoting factor RpfC	0.7541	3E-22
Rv3588c	canB	carbonic anhydrase	0.7582	8E-13
Rv1471	trxB1	thioredoxin TRXB1	0.7656	4E-07
Rv1815		Hypothetical protein	0.7814	2E-11
Rv0033	acpA	acyl carrier protein AcpA	0.8437	0.0008
Rv2466c		Hypothetical protein	0.8714	1E-09

S1B Table: Primers used in this study

Primer	Sequence (5’ to 3’)
KO1	GTCGTGGACCAGGTGGGG
KO2	GATCCACTAGTTCTAGAGCGGACACAGACTCCATCTGTTGGGGGT
KO3	GGCGCGCCGGTACCTCGAGTTAGCTGACTGGGCAGTGGCTGG
KO4	CCAGCGCAGACGACCGT
KO5	CGCTCTAGAACTAGTGGATCC
KO6	ACTCGAGGTACCGGCG
Comp1	CATCACCATCATCACCACGTGACGTTGCGAGTCGTTC
Comp2	AAGAAGGAGATATACATATGCATCACCATCATCACCAC
Comp3	TCATCCTTGTAGTCTCATAGCCCACCGCTGAGATAC
Comp4	AAGAAGGAGATATACATATGACCGAGCCGTGGATAG
Comp5	TCGTCATCCTTGTAGTCGCGATGCTCGTCGAACAC
Comp6	GGATCATCGAGCCGAGAACAGAAATATTGGATCGTCGGCACC
Comp7	ACACATGACCAACTTCGATAACGTTCTCGGCTCGATGATCC
Duet1	CATCACCATCATCACCACATGACGTTGCGAGTCGTTCC
Duet2	TTCTGTTCGACTTAAGCATCATAGCCCACCGCTGAGA
Duet3	TAAGAAGGAGATATACATATGACCGAGCCGTGGATAG
Duet4	CGGGTGGCTCCATCCTGCGCGATGCTCGTCGAACACG
Duet5	CAGCAGCCTAGGTTAATTATTTTTCGAACTGCGGGTGGCTCCATCCTGC
Duet6	GTGGTGATGATGGTGATG
Duet7	TAATTAACCTAGGCTGCTG
Duet8	TGCTTAAGTCGAACAGAA
Duet9	ATGTATATCTCCTTCTTATACTTAAC
T2B1	TAAGAAGGAGATATACATATGCGGGGTTCTCATCATCATCATC
T2B2	GTCGTCATCCTTGTAGTCTCAATCCTCAATGTTGTGACGGATC
