# Supplementary material for: The Mycobacterium tuberculosis PE15/PPE20 complex transports calcium across the outer membrane
Source: PLoS Biol. 2022 Nov 28;20(11):e3001906. doi: 10.1371/journal.pbio.3001906 (PMC9731449; doi:10.1371/journal.pbio.3001906)
Supplement: Attachment — Submitted filename: Response to reviewers.docx [file pbio.3001906.s007.docx]

We thank the reviewers for their excellent suggestions and constructive comments. We now added new data on PE15/PPE20 binding to Ca^2+^, the PDIM status of mutant strains, additional controls, added to the discussion, added detail to the Methods, and provide all RNA-seq data. Please see replies to the comments in detail below:

**Reviewer 1:**

**Major points**

1) Cellular localization:

“The authors do not describe the cellular fractionation method…”, “The authors show some Western blot data in Fig. S3, though the legend is superficial (abbreviations not explained) and the controls with markers for cellular localization appear to be lacking”. “Further, the authors do not prove that FLAG-tagged PE20 is functional.”

*We included a description of the fractionation method in Materials and Methods (lines 468-478). We also added detail to the legend of Fig. 4A to explain the abbreviations and controls used. The same cell fractions were used in Fig. 4A and Fig. S3A, as mentioned in the Figure S3 legend (“The same cell fractions as in Fig. 4A were used, see controls therein”). We know that the FLAG-tagged PPE20 is functional because the strain used in this experiment is the same we used for genetic complementation experiments in which FLAG-tagged PPE20 functionally complements ppe20 deletion in all three assays (ATP consumption, biofilm, Ca^2+^ influx, Fig.4 B,C,D,G).*

“The authors should extend discussion part of the manuscript. Several proteomic studies.” “Did authors analyze culture filtrate fraction by Western?

*We thank the reviewer for the references and extended the Discussion to include results from existing proteomic studies on PE15/PPE20 (lines 229-234). We did not test for PE15/PPE20 in culture filtrate, and previous proteomic results are contradictory. Several PE/PPE proteins, including PE15/PPE20 have been detected in the cell wall and in the CFP, but not consistently. The functional significance of this dual localization is unclear.*

2) Is PE15/PPE20 a channel?

“PPE20 purified alone from the cytosol of E. coli?”

*We did not purify either protein by itself. As the reviewer correctly notes, PE/PPE proteins are refractory to individual purification. We clarified that we purified and used the complex for experiments even if only PPE20 is shown, as in Figure 3C,D, and E (Lines 126-129). See also Methods line* 382 ff.

“…a positive control of a mutant that is indeed deficient in Mg2+ import (and thus showing a phenotype) is lacking.”

*Lacking a specific Mg^2+^ import mutant, and because it is a relatively minor point, we removed the statements about selectivity.*

3) Thermal melting assay

It is surprising to see that the thermal melting assays was done for PPE20 and PE15 as separately purified proteins.

*We co-purified PE15 and PPE20 for all biochemical experiments. We clarified that point (see also point 2 above).*

“the thermal melting assay only seemed to give some results for PPE20 alone, and not for PE15”

*PE15 did not produce interpretable results in this assay, as mentioned in line 146. We clarified in the Fig. 3 legend that the complex was used although only PPE20 is detected by Western blot and shown in Figure 3C.*

“…the results are counter-intuitive… How can the authors be sure that the presence of Ca2+ does not simply lead to more protein precipitation (via rather unspecific interactions) at elevated temperatures? Some positive controls with bona fide calcium binding protein in the same thermal melting setup would have helped to clarify this.”

*The effect of Ca^2+^ on PPE20 is somewhat counterintuitive, although not unprecedented. Proteins can be stabilized or destabilized by ligand binding, and a recent proteome-wide study on the basis of thermal shift analysis showed that ~17% of proteins were destabilized by ligand (ATP). For a channel in particular, ligand binding might be expected to be coupled to protein relaxation in the process of channel opening, which could well translate to lower thermal stability. We added the positive control showing the behavior of a known Ca^2+^ binding protein (new Fig. S2A). In addition, we included a negative control showing that Ca^2+^ does not generally increase protein denaturation (Fig. S2B). We think that this control addresses the reviewer’s concern more directly.*

If the authors want to stick to their claims regarding Ca2+ binding to PE15/PPE20, they have to perform additional assays (e.g. equilibrium dialysis or ITC) with the entire PE15/PPE20 complex. Further, they have to show that PE15/PPE20 forms a proper oligomeric protein that is membrane bound and reasonably behaved on size exclusion chromatography, when expressed in and purified from E. coli.

*We thank the reviewer for suggesting equilibrium dialysis as another, orthogonal assay to detect Ca^2+^ binding. We now show an equilibrium dialysis experiment that confirms Ca^2+^ binding by the PE15/PPE20 complex. Please see the new Fig. 3F. and G. and lines 148-154 (Results) and 423-437 (Methods).*

*The PE/PPE proteins are generally difficult to express and purify recombinantly, likely due to the typically large intrinsically disordered regions. Also, the yield of PE15/PPE20 when expressed in E. coli was very low so that we were not able to detect the complex by SEC. However, data in Fig. 3 conclusively show that PE15 and PPE20 bind.*

4) RNA-seq data

The authors should include a table with all other identified genes that are potentially involved in calcium homeostasis

*We provided all other significant differentially expressed genes in the new Table S1.*

**Minor points:**

1) “what is the binding affinity of the Ca sensor?”

*We added the Ca^2+^ binding affinity of Twitch-2B (K_D_: 200nM) in line 177.*

2) Figure 4D: “one would expect a drop in FRET signal after EGTA addition… Can the authors explain?”

*We do see a clear drop in FRET signal after EGTA addition, in particular in 7H9 medium (black versus red line, Fig. 5B). Given the high affinity of Twitch-2B for Ca^2+^ (200nM), however, it is not surprising that the drop is not more pronounced, as intracellular Ca^2+^ is expected to be tightly bound to Twitch.*

3) The experiments showing outer membrane localization of PE15/PPE20 are very important, but results of these experiments (western-blot and FRET) are shown in supplementary figures. They should be transferred/integrated into the main Figures.

*We agree and moved Figure S3A to the main Figures as Figure 4A.*

4) Line 166: the authors claim that the assay did not work in 7H9 due to low Ca2+ concentration in this medium. Why did the authors not just add a bit more calcium to show whether this claim holds true?

*7H9 is not a suitable medium for these experiments because the baseline Ca^2+^ concentration is too high, not too low (see Fig. 5B, grey versus black line). Adding more Ca^2+^ to 7H9 medium resulted in precipitation, probably due to its interaction with phosphates. Our use of “low” in this context was confusing, we changed the wording of this sentence (line 180-181).*

5) Line 183: more detailed description on cellular fractionation and subsequent anti-FLAG Western needed here.

*We added more detail in the Materials section (lines 469-478).*

**Reviewer 2:**

A major concern regarding the importance of the data: there are considerable technical challenges in generating Ca2+ depleted media. This is clear in that M. tuberculosis seems to be unaffected by Ca2+ in the medium - similar growth seems in Ca2+-free media to media with up to 10mM Ca2+ (Fig. S1). This raises a concern about the physiological relevance of the data (mammalian cells have intracellular Ca2+ of 0.01-0.1mM, extracellular free Ca2+ is around 1mM).

*If we correctly understand this comment, the reviewer is unconvinced that we fully and reproducibly depleted Ca^2+^ from medium based on a lack of an effect of Ca^2+^ on in vitro growth. We tested for baseline Ca^2+^ levels and depletion in media by inductively coupled plasma optical emission spectrometry and added these data showing precise quantitation of Ca^2+^ in medium (see new Fig. S1B).*

The role of PE15/PPE20 in Ca2+ acquisition may be clearer if the authors ensure that the PDIM layer is intact. Specifically, there is a technical issue: The authors use Tween80 as a detergent. Tween-80 partially strips the outer cell wall of M. tuberculosis resulting in shedding of PDIM and PE/PPE proteins. Tyloxapol is a somewhat milder detergent. Some of the experiments would possibly show clearer phenotypes by use of Tyloxapol.

*We share the concern about PDIM, as PDIM loss is common in in vitro culture. We analyzed the total lipids by thin layer chromatography and confirmed the presence of PDIM in all three strains (Fig S3C, lines 197-200). We repeated experiments with Tyloxapol and did not see differences to Tween-80. We nonetheless now show the Tyloxapol data (Fig 5D).*

The authors could increase the impact of their work be exploring the role of PE15/PPE20 during pathogenesis of resting versus activated bone marrow macrophages where Ca2+ fluxes of the host cell play a role in host responses.

*We agree. In vivo or macrophage experiments are a logical next step to fully characterize the function of PE15/PPE20, but we think it is beyond the scope of this manuscript. The main contribution of this paper is the identification of channel function of a PE/PPE protein pair that extends the novel channel paradigm for these proteins. These data support that transport might indeed be a shared function of the entire PE/PPE family with 169 members.*

Minor:

The authors should consider citing Sharma et al (2021)

*We cited the paper.*

Are there Ca2+ binding motifs in PPE20?

*We did not detect canonical Ca^2+^ binding motifs in PPE20.*

RNAseq data may need to be deposited in a public database.

*RNA-seq data have been deposited to NCBI - GEO accession GSE214266*

In its current state, the work is somewhat incremental

*The function of the large PE/PPE protein family of Mtb has been one of the most longstanding and perplexing puzzles in Mtb biology. For more than 20 years, speculation about their potential role, for example in antigenic variation, abounded but no conclusive evidence for this or another shared function emerged. A recent landmark paper then conclusively showed that a subset of the PE/PPE proteins function as nutrient channels (Wang et al., Science 2020). However, whether transporter function is a general function of the family of 169 PE/PPE proteins remains untested. Our PE/PPE pair is associated with a different type VII secretion system (Esx-3) and belongs to a different subfamily than the previous examples, suggesting a shared function across families and perhaps even all of these proteins. Given the intense interest and many false leads that have plagued the identification of PE/PPE function in the last 20 years, the difficulty of working with them biochemically, as well as the almost complete absence of understanding of Ca^2+^ homeostasis in Mtb, we do not consider our work incremental. We reworded parts of the Abstract to emphasize the importance of the PE/PPE proteins to understanding nutrient transport in Mtb.*

**Reviewer 3**

My only slight concern is the meaning attached to the "biofilm" assays. It is never very clear to me that this is anything more than formation of a surface pellicle and general hydrophobicity of the mycobacterial cells.

*We fully agree that Mtb biofilms remain poorly defined. However, the term biofilm as used in our study has already found its way into the literature and we would rather not cause confusion by calling the same phenomenon by a different name. Whatever the term used, we do not suggest any other relevance other than it being a Ca^2+^-dependent phenotype that serves as one of several tests to parse PE15/PPE20’s role in Ca^2+^ homeostasis.*

Cross-consultation comments:
We agree with the concerns of reviewer#2 that the role of PDIM and use of detergent should be looked at more closely.

*We tested the roles of PDIM and detergent, see reviewer 2.*

Likewise, the paper would strongly benefit from some further insights into the potential physiological role of PPE20/PE15 in calcium homeostasis.

*We show PE15/PPE20 function in the transport of Ca^2+^ and the first Ca^2+^-related cellular phenotypes in Mtb. Testing the role of the complex in an infection model is outside of the scope of this manuscript and mouse infection experiments would take many months and would likely be intractable because of the expected extensive redundancy among the 169 PE/PPE proteins.*
